# Evolutionary dynamics of HIV-1 subtype C in Brazil

**DOI:** 10.1038/s41598-021-02428-3

**Published:** 2021-11-29

**Authors:** Bernardino Souto, Vera Triunfante, Ana Santos-Pereira, Joana Martins, Pedro M. M. Araújo, Nuno S. Osório

**Affiliations:** 1 Life and Health Sciences Research Institute (ICVS), School of Medicine, University of Minho, Braga, Portugal; 2 ICVS/3B’s - PT Government Associate Laboratory, Braga, Guimarães, Portugal; 3 Department of Medicine, Federal University of São Carlos, São Carlos, Brazil

**Keywords:** HIV infections, Phylogeny

## Abstract

The extensive genetic diversity of HIV-1 is a major challenge for the prevention and treatment of HIV-1 infections. Subtype C accounts for most of the HIV-1 infections in the world but has been mainly localized in Southern Africa, Ethiopia and India. For elusive reasons, South Brazil harbors the largest HIV-1 subtype C epidemic in the American continent that is elsewhere dominated by subtype B. To investigate this topic, we collected clinical data and viral sequences from 2611 treatment-naïve patients diagnosed with HIV-1 in Brazil. Molecular epidemiology analysis supported 35 well-delimited transmission clusters of subtype C highlighting transmission within South Brazil but also from the South to all other Brazilian regions and internationally. Individuals infected with subtype C had lower probability to be deficient in CD4^+^ T cells when compared to subtype B. The HIV-1 epidemics in the South was characterized by high female-to-male infection ratios and women-to-child transmission. Our results suggest that HIV-1 subtype C probably takes advantage of longer asymptomatic periods to maximize transmission and is unlikely to outcompete subtype B in settings where the infection of women is relatively less relevant. This study contributes to elucidate factors possibly underlying the geographical distribution and expansion patterns of the most spread HIV-1 subtypes.

## Introduction

Retroviruses such as HIV (Human Immunodeficiency Virus) have an extreme capacity to generate genetic diversity^1^. HIV genetic diversity spectrum is divided into types I and II, with HIV-1 comprising the groups M, O, N and P. The pandemic group M is increasingly diversifying and comprises at least 10 subtypes, several sub-subtypes and recombinant forms^2,3^. Interestingly, these HIV-1 clades might be evolving at different rates, to modulate virulence^4,5^. Most accepted theories on virulence evolution postulate that the selection for an optimal virulence level follows a complex trade-off between the factors influencing pathogen induced-host mortality and between-host transmission^6^. In fact, M group subtypes were associated to differences in disease progression^7–11^, preferential transmission routes^12,13^ and different capacity to evade the immune system^14,15^ or therapy^16–18^. These differences possibly result in subtype-related advantages in different niches contributing for the global subtype spread dynamics^5,11^.

Subtype C causes nearly all infections in Southern Africa, Ethiopia and India being responsible for almost half of the HIV-1 infections in the world^19–21^. Despite the increasing amount of evidence that supports the geographic expansion of C subtype and other non-B subtypes in different continents^22–25^, globally, in the last decades, subtype C has been shown to have a decreasing profile, along with other subtypes, contrasting with subtype B^20^. In fact, subtype B remains the most geographically spread HIV-1 subtype worldwide. Ex vivo evidence following viral infection of peripheral blood mononuclear cells suggests that C subtype might be less cytopathogenic due to a preference for CCR5 co-receptor expressing cells and less fit when compared to B^26–28^. Furthermore, it was shown that HIV-1 subtype C is associated with slower rates of CD4^+^ T-cell declines and higher frequencies of long-term non-progression when compared to subtype A or D in women from Uganda and Zimbabwe^29^. In cohorts from Kenya^13^ or Tanzania^12^ it was found that pregnant women infected with subtype C had higher risk of mother-to-child transmission when compared with the ones infected with A or D.

Studies comparing in detail subtype C and B infections in human cohorts are limited by the rarity of informative clinical settings where subtype C and B co-exist in large numbers. In case of Brazil, the HIV-1 epidemics is dominated by B subtype. However, subtype C represents the most prevalent subtype in the South region of the country. The fact that most subtype C sequences from this region branch within a monophyletic clade suggest that this epidemic possibly initiated by the introduction in South Brazil, around 1960–80 s, of a single founder lineage derived from the radiation of an East African regional-specific group^30–35^. Reports show that in the early 2000s, C subtype represented around 30% of the HIV-1 infections in several cities in this region and that, after 2005, it became the most prevalent subtype, representing more than 40% of the cases^30,36^. Most strikingly, the spread of C subtype in other regions of Brazil outside of the South were revealed to be slow and modest. Despite intense and regular movement of people between the South and South-East regions, the South-East region of Brazil and other regions bordering South Brazil in Argentina, Paraguay or Uruguay remained with low subtype C prevalence^36–39^. The reasons underlying these regional differences are elusive and gaining insights into the introduction and regional expansion of HIV-1 subtype C in Brazil might give important information about C versus B subtype-related differences in what regards to within-host replication, virulence, transmission, and overall host population infection dynamics. Thus, in the present study, we investigated the phylogeography of HIV-1 lineages and compared clinical and epidemiological information from 2611 Brazilian patients.

## Results

### Proportion of HIV-1 subtype C infections in Brazil

**Figure 1:**
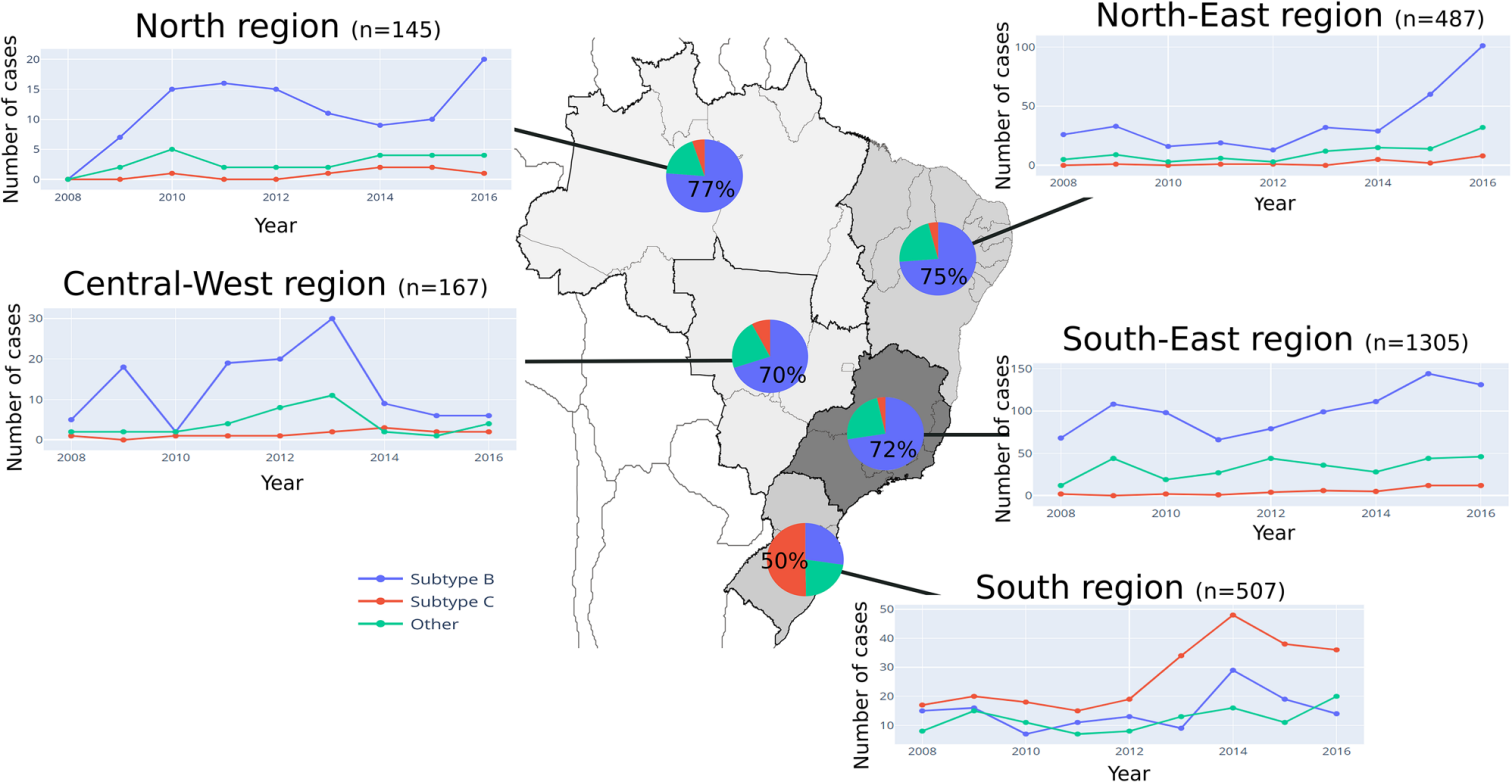
HIV-1 subtype distribution in Brazil nation-wide data obtained from 2008 to 2017 (n = 2611). (**A**) The pie charts represent the prevalence of subtypes B (blue), C (red) and other subtypes and recombinants (green) in the five macro-regions of Brazil. The shade of grey indicates the prevalence of HIV-1 (all subtypes) in each region (raging from the highest prevalence in dark grey to the lowest prevalence in light grey). (**B**) Absolute number of HIV-1 cases of subtype B, C and other subtypes in all Brazilian regions per year. Inkscape 0.92.4 (https://inkscape.org/) was used to create the map.

To investigate the differences in the proportion of cases caused by HIV-1 C and B subtypes in Brazil, we subtyped the sequences from all individuals that were treatment naïve and sampled from 01/2008 to 04/2017 at the National Genotyping Network of Brazil (n = 2611; Table S1). The region with the higher proportion of naïve HIV-1 infected individuals was the South-East (n = 1305; 49.98%) followed by the South (n = 507; 19.42%) and North-East (n = 487; 18.65%) (Fig. 1). HIV-1 subtype B was the most common at the country level with a total of 1675 cases, representing 64.15% of all infections in the studied population. Combining all regions, the proportion of C subtype among our sample was 13.02% (340 cases of a total of 2611). However, in the South, subtype C represented 50.30% of the cases being the most frequent in the region (Fig. 1). The analysis of the number of cases per year highlights that subtype C was consistently the most abundant in the South during the period under analysis (Fig. 1). In the South-East, the region with most HIV-1 infections, the number of cases with subtype C in the studied population never reached more than 12 cases per year, contrasting with the South, in which the number of C infections was superior to 30 cases per year in the period between 2013 and 2016, with a peak of 48 cases in the year of 2014 (Fig. 1). Overall, the South had a high growth in the number of cases caused by subtype C and was the only region where this subtype was more frequent than subtype B (Table S2).

### Subtype C associates with lower deficiency in CD4^+^ T cells when compared with B

**Figure 2:**
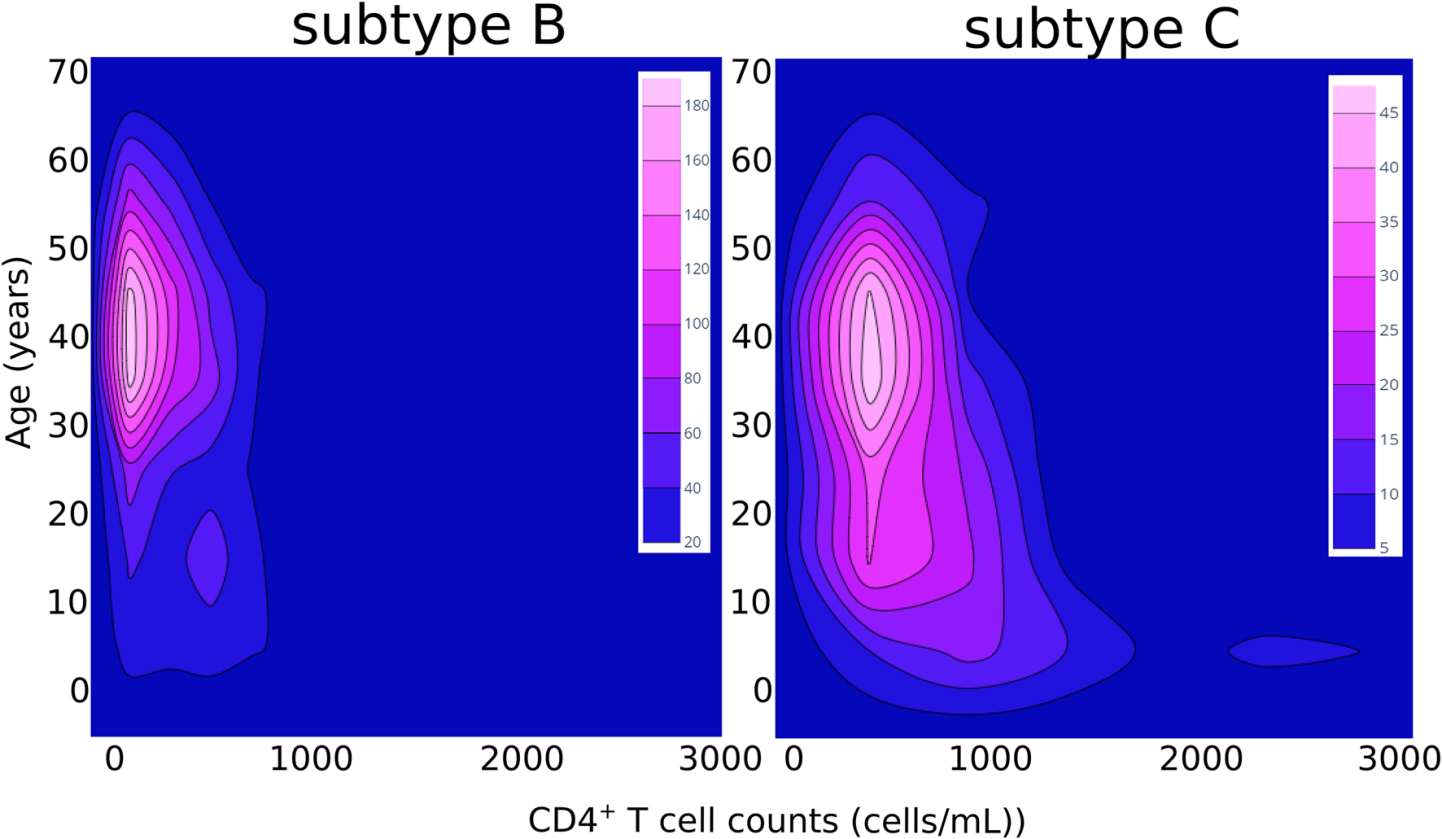
Age and CD4^+^ T cell counts in individuals infected with HIV-1 subtype B or C. The 2D contour histograms represent the Age (years) in the y axis and CD4^+^ T cell counts (cells/mL) in the x axis for all studied cases of HIV-1 subtype B infection (n = 1675) and subtype C infection (n = 340). The color scale represents the frequency of cases from blue (lower) to pink (higher).

To address subtype-related differences in infection progression outcomes, we compared the viral loads and CD4^+^ T cell counts between the infections caused by subtypes C or B. We found no statistically significant differences when comparing viral loads between individuals infected with C vs. B subtypes (*p* = 0.79). To test if individuals with very high viral loads could be confounding the analysis, we separated individuals with viral loads ≤ 100,000 virus/mL (n = 1927) from those with viral loads > 100,000 virus/mL (n = 535). The cut-off point of 100,000 virus/mL was chosen considering previous literature demonstrating its value to predict disease progression or treatment failure^40–42^. Again, we found no significant differences between C and B subtypes (*p* = 0.63; Table S3). To investigate the effect of HIV-1 subtype in CD4^+^ T cell counts we compared individuals with or without immunodeficiency and, among the immunodeficient, the ones with moderate or severe levels. Considering the age-related differences of CD4^+^ T cell normality, the classification of each case was done by adjusting the reference values according to the age of the subject (Table S4). The criteria used to define immunodeficiency accounted only for CD4^+^ T cell counts and was based on cut off points based on previous literature and commonly used in clinical practice^43,44^. Individuals infected with subtype C had a significant lower probability to be immunodeficient (*p* = 0.000) when compared with subtype B (Fig. 2; Table S5). This association was maintained when dividing the group by age (Table S5). Among the individuals with immunodeficiency, the ones infected with subtype C had significant lower probability of severe immunodeficiency (*p* = 0.001; Fig. 2; Table S5). Individuals with less than 18 years infected with subtype C had an even lower probability of severe immunodeficiency (*p* = 0.008; Table S5). Moreover, we decided to evaluate the proportion of ambiguous sites (PAS), a surrogate of age of infection^45–48^, on all the viral sequences and no significant difference was found between subtypes (Table S6). Overall, these results suggest that C subtype viruses, despite reaching viral loads similar to subtype B, are less able to cause a deficiency in CD4^+^ T cells, which could lead to longer asymptomatic periods and possibly increase the opportunity for transmission in some settings.

### Evidence for interregional and international subtype C transmission

**Figure 3:**
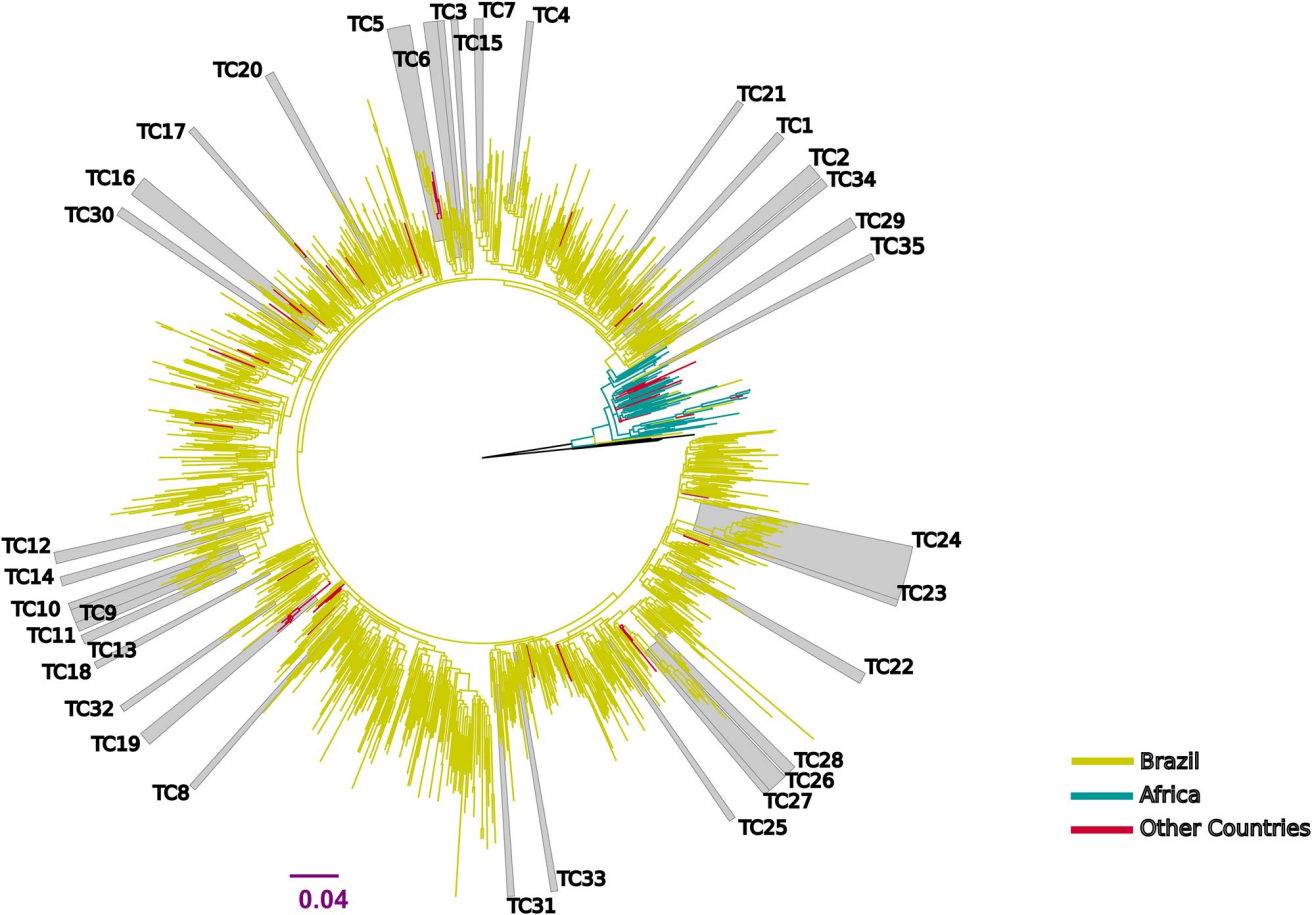
Maximum likelihood tree of the HIV-1 subtype C pol sequences (~ 1000 nucleotides) isolated in Brazil from treatment naïve individuals from 2008 to 2017 (n = 340) and closely related sequences from databases (total n = 1194). Multiple sequence alignments were performed using MAFFT v7.309 removing columns containing at least 10% gaps. Branch colors indicate the geographical origin of the sequences. Most subtype C sequences isolated from patients in Brazil are monophyletic (SH-like branch support 0.94) suggesting one single major founder event. Gray boxes indicate the positions of strongly supported clades (SH-aLRT ≥ 0.95). The branch lengths are drawn to scale with the bar at the bottom indicating nucleotide substitutions per site. Tree was rooted using HIV-1 subtype A1 reference sequences (black branches).

To gain insights into the transmission of subtype C in Brazil, we performed maximum likelihood (ML) and Bayesian phylogenetic analysis of the 340 subtype C sequences described in this study and 854 closely related sequences obtained from public databases (total n = 1194). The phylogenetic representation (Fig. 3, Table S7) demonstrated that the vast majority (99.26%; 1076 out of 1084) of the C subtype viruses isolated in Brazil were included in a monophyletic clade (SH-like branch support 0.94) that was nested with sequences from the East African region. This large clade also included sequences obtained from public databases and isolated in Asia, Europe, and other American countries. We then performed the characterization of transmission clusters and found 35 well-delimited transmission clusters (TC1 to TC35, Table 1) involving a total of 174 sequences. The average number of sequences per cluster was 4.97. TC24 was the largest cluster including a total of 24 sequences isolated in the South-East, Central-West or North regions of Brazil. Most of the clusters (18 out of 35, 51.43%) were exclusively formed from sequences isolated in the South of Brazil. From the nine clusters spanning more than one Brazilian region (Table 1, interregional), only clusters TC24 and TC35 did not include sequences isolated in the South. Interestingly, TC35 branched outside the diversification of the major founding event of subtype C in Brazil (Fig. 3) suggesting that rare transmission events of subtype C viruses from different introductions might exist in some parts of the country. Furthermore, we found four clusters that included sequences isolated outside Brazil (Table 1, international). The two largest (TC5, TC16) included sequences from the South, South-East, one other Brazilian region and other countries (USA, Spain, Portugal, or Germany). Most of the sequences in clusters (71.26%, 124 out of 174) lacked information on the reported route of infection. Among those with available data, transmission between heterosexual (28 cases) and MSM (12 cases) were the most reported. The estimates for the time of the MRCA for each cluster ranged from 1992 to 2009. The cluster depth analysis (obtained for each cluster by subtracting the most recent sampling date minus the time of MRCA) supports that six transmission clusters (TCs 2, 5, 16, 22, 24 and 26) were ongoing for more than 20 years (Table 1).

**Table 1:** Characterization of the 35 transmission clusters of HIV-1 subtype C virus identified in this study.

Cluster	Number of individuals					Place of sampling (n)							Sampling date range	Time of MRCA (95% HPD, years)	Cluster depth (years)
						Brazil					Other	Missing			
	Total	Male	Female	N/A		N	S	NE	SE	CW					
TC5	10	6	1	3	International	0	1	1	5	0	3^a^	0	2009.0–2017.2	1992.7 (1984.8–1999.6)	24.5
TC16	9	5	0	4		0	4	0	2	1	2^c^	0	2008.0–2017.1	1996.3 (1990.6–2001.4)	20.8
TC19	6	0	0	6		0	0	0	0	0	6^b^	0	N/A	N/A	N/A
TC17	3	1	1	1		0	2	0	0	0	1^d^	0	2007.0–2017.2	1998.5 (1993.1–2003.3)	18.7
TC24	24	16	4	4	Interregional	3	0	0	13	8	0	0	2010.0–2017.2	1992.9 (1985.7–1999.7)	24.3
TC26	9	4	1	4		0	5	0	3	0	0	1	2006.0–2017.0	1995.9 (1989.4–2001.8)	21.1
TC9	6	2	4	0		1	1	0	1	3	0	0	2010.0–2017.0	1997.1 (1991.4–2002.4)	19.9
TC29	5	2	2	1		0	4	0	0	1	0	0	2008.0–2014.8	1999.7 (1994.7–2004.1)	15.1
TC34	4	1	2	1		0	1	0	3	0	0	0	2013.0–2016.8	2003.2 (1997.7–2008.5)	13.6
TC31	3	0	3	0		0	1	2	0	0	0	0	2016.7–2016.8	2000.9 (1994.9–2006.7)	15.9
TC27	3	0	3	0		0	2	0	1	0	0	0	2016.1–2016.2	2008.5 (2002.2–2013.5)	7.7
TC32	3	0	3	0		0	2	0	1	0	0	0	2016.2–2016.8	2002.5 (1997–2008.3)	14.3
TC35	3	0	1	2		0	0	1	1	1	0	0	2006.0–2016.7	1999.5 (1995.3–2003.6)	17.2
TC2	7	2	1	4	Regional	5	0	0	0	0	0	2	2004.0–2016.8	1992.2 (1982.3–2000.6)	24.6
TC6	6	1	2	3		0	6	0	0	0	0	0	2012.0–2015.5	1996.3 (1990.1–2002)	19.2
TC12	5	1	0	4		0	5	0	0	0	0	0	2009.8–2015.0	2006.9 (2003.1–2009.8)	8.1
TC22	5	2	2	1		0	5	0	0	0	0	0	2008.8–2015.9	1995.5 (1988.6–2001.7)	20.4
TC28	5	5	0	0		0	5	0	0	0	0	0	2013.2–2017.2	2004.9 (1999.8–2009.7)	12.3
TC7	4	0	4	0		0	4	0	0	0	0	0	2013.3–2017.1	2000.9 (1995.3–2006.1)	16.2
TC1	4	3	0	1		0	4	0	0	0	0	0	2008.0–2016.9	2000.2 (1995.3–2004.8)	16.7
TC13	4	0	4	0		0	4	0	0	0	0	0	2012.6–2014.5	1999.8 (1993.9–2005.3)	14.7
TC14	4	2	2	0		0	4	0	0	0	0	0	2011.3–2015.5	1999.8 (1993.7–2005.2)	15.7
TC11	4	1	3	0		0	4	0	0	0	0	0	2015.9–2017.3	1999.2 (1993.2–2005.2)	18.1
TC30	4	1	1	2		0	4	0	0	0	0	0	2008.0–2014.4	1999.3 (1994.3–2003.8)	15.1
TC20	4	0	4	0		0	4	0	0	0	0	0	2008.4–2015.3	1998.1 (1992.3–2003.5)	17.2
TC21	3	1	2	0		0	3	0	0	0	0	0	2012.2–2013.2	2000.9 (1995.1–2006.5)	12.3
TC33	3	2	1	0		0	3	0	0	0	0	0	2014.4–2015.6	2001.3 (1995.6–2007.1)	14.3
TC4	3	1	2	0		0	3	0	0	0	0	0	2013.8–2016.7	2009.5 (2004.8–2013.2)	7.2
TC15	3	1	1	1		0	3	0	0	0	0	0	2008.0–2012.2	1998.8 (1993.4–2004.1)	13.4
TC18	3	0	3	0		0	3	0	0	0	0	0	2011.9–2014.2	2009.1 (2005.4–2011.8)	5.1
TC10	3	1	2	0		0	0	0	3	0	0	0	2010.4–2014.1	2006.7 (2002.6–2010)	7.4
TC8	3	2	1	0		0	3	0	0	0	0	0	2015.8–2017.2	1998.8 (1992–2005.5)	18.4
TC23	3	0	3	0		0	3	0	0	0	0	0	2013.4–2014.8	2001.3 (1995.7–2006.5)	13.5
TC3	3	0	0	3		0	0	0	2	0	0	1	2007.0	2002.1 (1998.4–2005.4)	4.9
TC25	3	0	2	1		2	0	0	0	0	0	1	2010.0–2010.4	1998.8 (1992.4–2004)	11.6

^a^USA (1), Spain (1), Portugal (1).

^b^UK (6).

^c^Germany (1), Spain (1).

^d^Germany (1).

### Relevant role of the South-East in the transmission of subtype C

**Figure 4:**
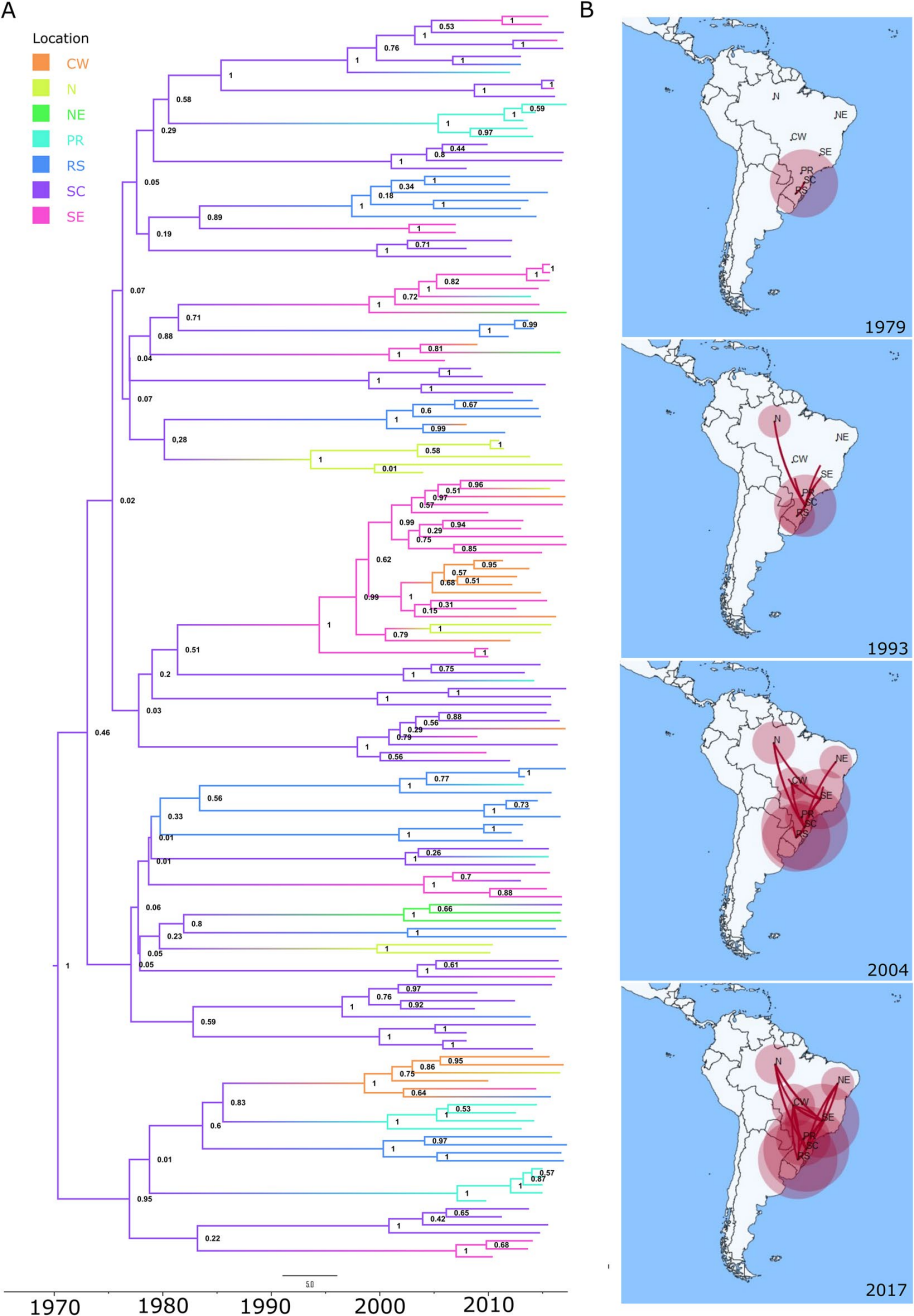
Phylogeographic analysis of the evolution of HIV-1 subtype C transmission clusters. (**A**) Bayesian MCC time scaled discrete phylogeographic tree built using BEAST v1.10.4 of the HIV-1 subtype C sequences included in the transmission clusters that were sampled in Brazil and have complete information (n = 156). Tip location was defined as Brazilian region or state of sample collection. (**B**) Geographical representation of this transmission history. Acronyms and number of sequencies per location: N (North region of Brazil, n = 11), NE (North-East region of Brazil, n = 4), CW (Central-West region of Brazil, n = 14), SE (South-East region of Brazil, n = 34), SC (state of Santa Catarina in the South region of Brazil, n = 43), RS (state of Rio Grande do Sul in the South region of Brazil, n = 31), and PR (state of Paraná in the South region of Brazil, n = 19). SpreaD3 v0.9.6 (https://rega.kuleuven.be/cev/ecv/software/SpreaD3) was used to visualize the phylodynamic reconstruction resulting from Bayesian inference.

To assess the viral diffusion patterns, a phylogeographic analysis (Fig. 4A) was performed considering the sequences included in the transmission clusters that were sampled in Brazil and have complete information (n = 156). Tip location was defined as Brazilian region of sample collection (North, North-East, Central-West or South-East). Due to larger sample size, sequences from the South region were classified according to the state of origin (Santa Catarina (SC), Rio Grande do Sul (RS), or Paraná (PR)). Analysis with TempEst showed a positive correlation between genetic divergence and sampling time (r = 0.45, R^2^ = 0.20) suggesting that the temporal signal of the dataset was suitable for phylogenetic molecular clock analysis^49^. The root of subtype C epidemic in Brazil was inferred to SC (psp = 0.93, pp = 1.00) in the South. Transmission clusters including C sequences from outside of the South were estimated to have a time of MRCA as early as 1993 (1983.8–2001.9) with the regions of the South-East and North, having the highest probability (psp = 0.85, pp = 1.00 and psp = 0.83, pp = 1.00, respectively) of being the first points of introduction from the South into other regions (Fig. 4B). A total of 5 pairwise rates of diffusion between locations were found to have a strong support value (Bayes factor (BF) > 10). These include the transmission among SC and the South-East (BF = 34,202.74, pp = 1.00), the South-East and Central-West (BF = 1898.13, pp = 1.00), SC and RS (BF = 58.83, pp = 0.96), SC and PR (BF = 37.50, pp = 0.95), or RS and Central-West (BF = 11.58, pp = 0.84). Furthermore, the linkage between South-East and North, or South-East and North-East was supported by a BF above 3. The results suggest that, at a given point in the transmission history, the South-East not only received C viruses from the South but was also involved in transmission to other Brazilian regions. These findings were also supported by a phylogeographic analysis using the transmission cluster sequences grouped by Brazilian state and including sequences sampled outside Brazil (n = 161, Fig. S1). Additionally, this analysis showed well supported diffusion rates (BF > 10) for the international transmission of the Brazilian C subtype clade relating the Southern state of RS with Germany and Spain with the United States of America (Table S8).

### Demographic differences in subtype C infections

Having established evidence for intense South to South-East transmission of subtype C, we then explored the demographic and epidemiologic characteristic of the HIV-1 epidemics in these regions to investigate possible reasons for the inferior capacity of C subtype to become dominant outside the South. In total, 60.18% (204 of 339) of the infections by subtype C in Brazil were in women and only 39.82% (135 of 339) in men (OR = 1.64; CI = 1.30–2.08; *p* = 0.000). In accordance, we found significant differences (*p* = 0.0160) in the distribution of the sex of the HIV-1 infected individuals in the South when compared with the South-East (Table 2). In the South, HIV-1 affected more females (55.82%) than males (female-to-male ratio = 1.27) while in the South-East most of the infections were in male (50.04%; female-to-male ratio = 0.98). Despite the missing data, within our study population, mother-to-child transmission was significantly more likely (CI = 1.76–6.96; *p* = 0.0002) to occur in the South than in the South-East. Moreover, the number of infected individuals with less than 18 years of age infected with HIV-1 in the South was also significantly (*p* = 0.000) higher than in the South-East. Transmission between men that have sex with men (MSM) was significantly more associated with the South-East (OR = 3.72; CI = 1.22–15.13; *p* = 0.0218). These findings highlight clear demographic and epidemiological differences between these two neighboring Brazilian regions.

**Table 2:** Epidemiological comparison of HIV-1 epidemics between South and South-East Brazil.

		Brazil	South	South-East	OR*	CI	*p* value
**Sex**							
Male		1314 (50.33%)	223 (43.98%)	653 (50.04%)	0.78	0.63–0.95	0.016
Female		1286 (49.25%)	283 (55.82%)	643 (49.27%)	1.29	1.05–1.58	
Missing		11 (0.42%)	1 (0.20%)	9 (0.69%)	0.28	0.01–2.06	0.359
**Age**							
< 18 yrs		702 (26.89%)	165 (32.54%)	276 (21.15%)	1.80	1.43–2.26	0.000
≥ 18 yrs		1903 (72.88%)	341 (67.26%)	1027 (78.70%)	0.55	0.44–0.70	
Missing		6 (0.23%)	1 (0.20%)	2 (0.15%)	1.29	0.02–23.78	1.000
**Transmission route**							
MSM		50 (1.91%)	4 (0.79%)	32 (2.45%)	0.27	0.07–0.82	0.022
Mother-to-child		82 (3.14%)	24 (4.73%)	25 (1.92%)	3.49	1.76–6.96	0.000
Heterosexual		165 (6.32%)	27 (5.33%)	82 (6.28%)	0.70	0.38–1.30	0.260
Other		12 (0.46%)	2 (0.39%)	7 (0.54%)	0.72	0.07–3.96	0.984
Missing		2302 (88.17%)	450 (88.76%)	1159 (88.81%)	0.99	0.72–1.38	0.974
**Subtype**							
B**	Male	887 (53.27%)	49 (59,04%)	189 (53.54%)	1.25	0.77–2.04	0.366
	Female	778 (46.73%)	34 (40.96%)	164 (46.46%)	0.80	0.49–1.30	
C***	Male	135 (39.82%)	69 (43.67%)	9 (50.00%)	0.78	0.28–2.12	0.609
	Female	204 (60.18%)	89 (56.33%)	9 (50.00%)	1.29	0.47–3.152	
Other	Male	292 (48.99%)	33 (54.10%)	52 (49.52%)	1.20	0.65–2.27	0.571
	Female	304 (51.01%)	28 (45.90%)	53 (50.48%)	0.83	0.44–1.57	
Missing		898 (34.39%)	254 (50.01%)	1018 (78.01%)	0.28	0.23–0.35	0.000
Total		2611	507	1305			

*The OR, CI and *p* value are presented for the South versus South-East comparison using two-tailed Fisher Exact Test or Corrected Mantel–Haenszel chi-square test were applicable.

**For the difference in the prevalence of subtype B between men and women in Brazil, the following statistical result was found: OR = 1.36, CI = 1.15–1.59, *p* = 0.000.

***For the difference in the prevalence of subtype C between women and men in Brazil, the following statistical result was found: OR = 1.64, CI = 1.30–2.08, *p* = 0.000.

## Discussion

HIV-1 subtypes C and B can be considered the evolutionarily most successful HIV-1 subtypes. Given the differences in geographic distribution between C and B subtypes it is reasonable to assume that there are particularities in these viruses possibly conferring subtype-specific advantages in different settings. In this study, country level clinical and demographic data, and partial sequences of the HIV-1 genome (pol sequence) originating from routine genotypic testing for resistance to antiretroviral therapy were investigated. The observed proportion of HIV-1 infections by Brazilian region in the study population was in accordance with the official HIV-1 prevalence reports^50^. The pol region, previously shown to be able to accurately reconstruct HIV transmission^51^, was used for phylogenetic analysis. Regarding HIV-1 subtype distribution in Brazil, our results update and expand to the country-level previous literature^52–54^ in showing that Brazil has bordering regions dominated in prevalence by subtype B or C. During the period under analysis, subtype C led in proportion only in the South with the rest of Brazil being dominated by subtype B. Most interestingly, despite intense and regular movement of people between the South and South-East regions, the lowest overall subtype C proportion of cases in the studied population was found in the South-East (3.52%; 44 cases out of 1248).

Subtype C was previously associated with higher CD4^+^ T cell counts in African cohorts when compared with subtypes A and D^29^. In the comparison with B subtype, our analysis in the Brazilian cohort suggests that subtype C, despite reaching similar viral loads than subtype B, could lead to more moderate rates of destruction of CD4^+^ T cells. In fact, among people infected with subtype C there were significantly less individuals with deficiency in CD4^+^ T cells when compared with the ones infected with subtype B, which was not due to differences on the age of the infected individuals or in the time since infection, as no significant differences were observed on the statistical analysis of the PAS^45–48^. This could lead to longer asymptomatic periods in subtype C infections and possibly increased opportunities for transmission. To investigate the C subtype transmission, we performed a molecular epidemiology and phylogeographical analysis using the 340 C subtype sequences obtained in this study and the closest related sequences from databases. This was performed to enrich the information that could be obtained related to the transmission outside Brazil. Our analysis generated information on the origin and probable place of introduction of C subtype in Brazil. In accordance with the previous studies^30,32^, we found strong evidence supporting one major founding event of introduction of subtype C in Brazil originating from Middle East African countries. We found no evidence supporting the introduction from UK to Brazil as suggested in one study^31^. We did find a transmission cluster (TC19) with sequences isolated in the UK that likely originated in Brazil and was transmitted to the UK. We found strong statistical support for international transmission from the Southern Brazil state of Rio Grande do Sul (RS) to Germany. This link is possibly explained by the known migratory fluxes between these two geographic locations.

In our analysis, the state with the highest probability for the place of entrance of subtype C in Brazil was Santa Catarina (SC). The characterization of transmission clusters and phygeographic dynamics suggests that the inferior capacity of C subtype to thrive outside the South was not due to absence of cross-regional transmission. In fact, we found that more than 20% of the C subtype transmission events bridged, in the last decades, the South and at least one other Brazilian region with emphasis on the South-East. We found strong statistical support indicating that the South-East region was not only recipient but also donor in interregional transmission clusters of subtype C viruses. This suggests that, although the South-East has among the lowest overall proportion and annual growth rate of subtype C in the country it played a role in disseminating C subtype virus to other Brazilian regions. Considering our results, it is tempting to speculate that for HIV-1 subtype C to thrive in a population it relies on its high within-host replicative capacity (like that of B subtype) but possibly also takes advantage of longer asymptomatic periods that might increase its opportunities to transmit. The epidemiological comparison between the South and South-East Brazil suggests that C subtype capacity to outcompete B might be facilitated in settings with higher female-to-male infection ratios and women-to-child transmission. However, these conclusions are limited by the presence of missing data on the reported route of infection and to what is possible by means of a cross-sectional study. Notwithstanding, this data finds parallels in previous studies in African cohorts showing that C subtype was more adapted to women-to-child transmission than A or D subtypes^12,13^. In a Kenyan cohort, it was found that pregnant women infected with subtype C were significantly more likely to shed HIV-1–infected vaginal cells than were those infected with subtype A or D^13^. Whether C subtype virus are present in higher levels in cells from the vaginal mucosa or even breast milk when compared to B subtype virus has not, to our best knowledge, been studied, being a matter for future investigation. On the other hand, the distribution of HIV infection among men, women and children is also influenced by sociocultural factors such as breast feeding and other gender equality-related factors. It is relevant to point out that the practice of cross-breastfeeding was a culturally established and accepted behavior in Brazil^55,56^. It was initially provided by lactating slaves mainly originating from the same African regions that are the most probable point of origin of the HIV-1 subtype C introduced in Brazil. Long after slavery was abolished and at least until the first half of the XX century, it was frequent that lactating Afro-Brazilian women were paid to cross-breastfeed^56^. It is possible that sociocultural heritages from this past influenced the introduction and transmission of subtype C and, consequently, its distribution in the Brazilian territory. The South has the highest prevalence in Brazil of AIDS in pregnant women and children and the higher female-to-male infection ratio^50^. The degree of genetic mixing in the Brazilian population is very high being unlikely that differences in human population ancestry between the South and the South-East could be the explanation for the high rate of subtype C infections. However, subtype C could have found in the Southern region of Brazil, sociocultural and behavioral conditions favorable to its dissemination with similarities to those found in African and Asian regions, where it is also the most prevalent HIV-1 subtype^20,57^.

Overall, this study opens lines of research on the differences between the two most prevalent HIV-1 subtypes and, at the same time, it is useful for the management of the health care and public HIV-1 control policies. Regarding the dynamics between B and C subtypes it is possible that C subtype outcompetes B only in settings with sizable infection of women and women-to-child transmission. Thus, it is suggested that, where the prevalence of subtype C is higher, care professionals and public policies define specific strategies for the protection of women and the pregnancy-puerperal cycle against HIV infection. Targeting this group by close surveillance to make the diagnosis and treatment as close as possible to the time of infection is likely to reduce the epidemiological burden of subtype C HIV-1 infections.

## Materials and methods

### Study population

Data was collected from HIV-1 infected patient records (n = 2611, Table S1) available at the Specialized Assistance Services on Sexually Transmissible Diseases and HIV/Aids including all 26 Brazilian states and Federal District from 01/01/2008 to 04/30/2017 that met the inclusion criteria for this study. The inclusion criteria were availability of partial HIV-1 genome sequence, and absence of previous antiretroviral treatment upon sampling. For all cases matching the criteria the following patient data was collected anonymously from previously available records: self-reported transmission route; sex; birth year; date of the viral sample collection for sequencing; CD4^+^ T-cell count at sampling; viral load at sampling; geographical origin of the sample; and pregnancy. The study was approved and was granted exemption from written informed consent by the Brazilian national ethic committee, "Comissão Nacional de Ética em Pesquisa (Conep)", through the protocol CAAE 53757016.0.0000.5504. All methods were performed in accordance with the Declaration of Helsinki.

### Sequencing

DNA sequencing, from the reverse transcriptase PCR amplicons was performed with commercially available HIV-1 genotyping systems based on Sanger sequencing and performed using standardized protocols in the National Genotyping Network of Brazil. The HIV-1 genome sequence portion used in this study corresponds to the pol region. The HIV-1 positions in this study refer to the HXB2 HIV-1 reference genome (GenBank: K03455.1). All multiple sequence alignments were performed using MAFFT v7.309^58^ removing columns containing at least 10% gaps. The HIV-1 subtype was assigned using Rega HIV-1 Subtyping Tool v3.0^59^, Comet HIV-1 v2.3^60^, jpHMM^61^, RIP v3.0^62^, SCUEAL^63^, and SNAPPY^64^. The results of the different tools were compared, and subtype was classified based on the agreement between the used tools and manual inspection of the results from phylogenetic and recombination analysis. The 2611 sequences selected for this study were made available in GenBank (accession numbers pending).

### Phylogenetic analysis

To obtain additional sequences from outside the National Genotyping Network of Brazil we queried the HIV reference sequence database (http://www.hiv.lanl.gov/) using BLAST^65^. For each of the 340 subtype C sequences described in this study the 10 most closely related generated outputs were selected. We excluded duplicates or sequences from the same patient and sequences showing evidence of recombination. Applying these criteria 854 database sequences were added to this study for phylogenetic analysis. An alignment of 1194 sequences was used to make a phylogenetic reconstruction using PhyML v3.0^66^. The best fitting substitution model was GTR + G4 + I, determined by PhyML SMS(Smart Model Selection) using AIC (Akaike Information Criterion)^67^.The heuristic trees search was performed using SPR and NNI methods. The branch support was calculated with the approximate likelihood-ratio (aLRT) SH-like test. The tree with the best likelihood value was performed using SPR with 3 random starting trees (Fig. 2). Bayesian evolutionary and phylogeographic analyses were performed using BEAST v1.10.4^68,69^, with GTR + G4 + I for two different codon partitions (1 + 2, 3), as nucleotide substitution model, coalescent Skygrid model and uncorrelated relaxed clock. The site model GTR + G4 + I corresponding to the best model selected by jModelTest program^70^. The sampling Brazilian region, state or country outside Brazil were used as discrete traits. A symmetric discrete traits substitution model selecting the option to infer social network with Bayesian Stochastic Search Variable Selection (BSSVS) method was used to estimate transition rates between locations. The temporal signal of the data was tested by TempEst^49^. Two different runs (random seeds) of 320 million generations, converged to similar values. Outputs were analysed with Tracer v1.7.1^71^ to ensure all parameters had an effective sampling size (ESS) superior to 200. The two multiple tree output files were combined, using LogCombiner v1.10.4^68^, to build the maximum clade credibility tree with mean heights with TreeAnnotator v1.10.4^68^. The resulting log files were also combined with LogCombiner v1.10.4^68^. The phylogeographic representations were created with SpreaD3^73^. For database sequences from outside Brazil, the country’s locations were plotted as their geographic centre. 

### Definition of transmission cluster and tree visualization

The criteria for the definition of a clade as a transmission cluster were likelihood ratio test (aLRT) SH-like branch support ≥ 0.95 (estimated with PhyML v3); branch posterior probability ≥ 0.99 (estimated with BEAST v1.10.4); mean cluster genetic distance < 0.003 substitutions per site; and maximum genetic distance < 0.05 substitutions per site. MEGA X v10.05^72^ was used for genetic distance calculation. Only clusters with more than 2 sequences were included. The phylogenetic tree shown in Fig. S1 was used for the characterization and dating of transmission clusters. FigTree v1.4.4 was used for visualization and manipulation of the trees^73^.

### Statistical analysis

After verifying and optimizing the quality of epidemiological data (transmission route; sex; birth year; date of the viral sample collection for sequencing; CD4^+^ T-cell count at sampling; viral load at sampling; geographical origin of the sample), they were organized into spreadsheets and processed by the software Epi Info, from the Center for Disease Control and Prevention (United States). For statistical analysis, the Mantel–Haenszel chi-square test was used when the minimum sample size in all variables was greater than or equal to 5. When sample size was less than 5 units in at least one of the variables, the Fisher exact test was used for calculating the Odds Ratio and the corrected Mantel–Haenszel chi-square test for calculating the *p* value. In all cases, the tests were two-tailed, and the level of significance considered was 5%.

## Supplementary Information

Supplementary Information 1.

Supplementary Video 1.
