# Supplementary material for: Evolutionary dynamics of HIV-1 subtype C in Brazil
Source: Sci Rep. 2021 Nov 29;11:23060. doi: 10.1038/s41598-021-02428-3 (PMC8629974; doi:10.1038/s41598-021-02428-3)
Supplement: Supplementary file 1 — Supplementary Information 1. [file 41598_2021_2428_MOESM1_ESM.docx]

**Evolutionary dynamics of HIV-1 subtype C in Brazil**

Bernardino Souto^1,2,3^, Vera Triunfante^1,2^, Ana Santos-Pereira^1,2^, Joana Martins^1,2^, Pedro M.M. Araújo^1,2^ and Nuno S. Osório^1,2^

^1^Life and Health Sciences Research Institute (ICVS), School of Medicine, University of Minho, Braga, Portugal.

^2^ICVS/3B’s - PT Government Associate Laboratory, Braga, Guimarães, Portugal.

^3^Department of Medicine, Federal University of São Carlos, São Carlos, Brazil.

**Supplementary information**

Table S1– Distribution by subtype and region of the studied 2611 samples.

| **Region** | **Subtype** | | | **Total** |
| --- | --- | --- | --- | --- |
|  | B | C | Other |  |
| Central-West | 117  70,06% | 13  7,78% | 37  22,16% | 167  6,40% |
| North-East | 364  74,74% | 18  3,70% | 105  21,56% | 487  18,65% |
| North | 111  76,55% | 7  4,83% | 27  18,62% | 145  5,55% |
| South-East | 946  72,49% | 47  3,60% | 312  23,91% | 1305  49,98% |
| South | 137  27,02% | 255  50,30% | 115  22,68% | 507  19,42% |
| Total | 1675  64,15% | 340  13,02% | 596  22,83% | 2611 |

Table S2 -Annual growth rate of HIV-1 infections in the Brazilian cohort under study by subtype and region.

| **Subtype** | **Region** | **Annual growth rate (%)** |
| --- | --- | --- |
| **C** | South-East | 0.67 |
|  | South | 1.73 |
|  | North-East | 0.66 |
|  | North | 1.29 |
|  | Central-West | 1.44 |
| **B** | South-East | -1.04 |
|  | South | -1.23 |
|  | North-East | -1.54 |
|  | North | 0.03 |
|  | Central-West | -1.35 |

Table S3 - Viral load comparison between individuals infected with subtypes C vs. B or C vs.other non-B and non-C subtypes.

| **Viral load**  **(copies/mL)** | **Subtype** | | | **N** | **C vs. B** | | | **C vs. other** | | |
| --- | --- | --- | --- | --- | --- | --- | --- | --- | --- | --- |
|  | B | C | Other |  | OR | CI | p value | OR | CI | p value |
| <=100,000 | 1270  65.91% | 251  13.03% | 406  21.07% | 1927 | 0.93 | 0.70-1.25 | 0.6319 | 1.26 | 0.91-1.76 | 0.0162 |
| >100,000 | 325  60.75% | 69  12.90% | 141  26.36% | 535 | 1.07 | 0.80-1.43 |  | 0.79 | 0.57-1.10 |  |
| Missing | 80  53.69% | 20  13.42% | 49  32.89% | 149 | 1.24 | 0.74-2.04 | 0.3919 | 0.70 | 0.40-1.18 | 0.1880 |

Table S4 – Age and CD4+ T cell count cut-off points* used for the classification of immunological status.

| **Immunological status** | **Age group** | | |
| --- | --- | --- | --- |
|  | **<1** | **1-5** | **>5** |
|  | **CD4+ counts (cell/mm^3^)** | | |
| Without immunodeficiency | >1500 | >1000 | >500 |
| Moderate immunodeficiency | 750-1499 | 500-999 | 200-499 |
| Severe immunodeficiency | <750 | <500 | <200 |

* The used cut-off points were based on previous literature ^39,40^

Table S5- Comparison of the number of infections with subtype C, B and other classified as leading to immunodepression with moderate or severe levels.

| **Age group**  **(years old)** | **Immunodepression** | **Subtype** | | | **N*** | **C vs. B**** | | | **C vs. Other**** | | | |
| --- | --- | --- | --- | --- | --- | --- | --- | --- | --- | --- | --- | --- |
|  |  | B | C | Other |  | OR | CI | p value | OR | CI | | p value |
| All | No | 562  60.30% | 150  16.09% | 220  23.61% | 932 | 1.71 | 1.33-2.19 | 0.000 | 1.36 | 1.02-1.81 | | 0.034 |
|  | Yes | 979  68.13% | 153  10.65% | 305  21.22% | 1437 | 0.58 | 0.46-0.75 |  | 0.74 | 0.55-0.98 | |  |
| < 18 | No | 178  51.30% | 66  19.02% | 103  29.68% | 347 | 1.68 | 1.05-2.70 | 0.029 | 1.02 | 0.60-1.74 | | 0.948 |
|  | Yes | 154  63.64% | 34  14.05% | 54  22.31% | 242 | 0.60 | 0.37-0.95 |  | 0.98 | 0.57-1.67 | |  |
| ≥ 18 | No | 384  65.64% | 84  14.36% | 117  20.00% | 585 | 1.52 | 1.11-2.05 | 0.007 | 1.51 | 1.06-2.16 | | 0.022 |
|  | Yes | 825  69.04% | 119  9.96% | 251  20.00% | 1195 | 0.66 | 0.49-0.90 |  | 0.66 | 0.46-0.94 | |  |
|  |  | | | | | | | | | | | |
| All | Yes. Moderate | 320  62.99% | 71  13.98% | 117  23.03% | 508 | 1.78 | 1.26-2.52 | 0.001 | 1.40 | 0.94-2.06 | 0.099 | |
|  | Yes. Severe | 659  70.94% | 82  8.83% | 188  20.24% | 929 | 0.56 | 0.40-0.79 |  | 0.72 | 0.48-1.07 |  |  |
| < 18 | Yes. Moderate | 61  56.48% | 22  20.37% | 25  23.15% | 108 | 2.78 | 1.29-6.20 | 0.008 | 2.11 | 0.87-5.24 | 0.094 | |
|  | Yes. Severe | 93  69.40% | 12  8.96% | 29  21.64% | 134 | 0.36 | 0.16-0.78 |  | 0.47 | 0.19-1.15 |  |  |
| ≥ 18 | Yes. Moderate | 259  64.75% | 49  12.25% | 92  23.00% | 400 | 1.53 | 1.03-2.26 | 0.033 | 1.21 | 0.77-1.89 | 0.403 | |
|  | Yes. severe | 566  71.19% | 70  8.81% | 159  20.00% | 795 | 0.65 | 0.44-0.97 |  | 0.83 | 0.53-1.30 |  |  |

* From the 2611 cases selected for this study 245 lacked information on age, or CD4+ counts and were not included in this calculation. The missing values had a random distribution between the groups.

**Statistical comparisons were performed with corrected Mantel-Haenszel chi-square test.

Table S6 – Comparison of proportion of ambiguous sites (PAS) between B and C subtypes.

|  |  | **B** | | **C** | | **C vs. B** | | |
| --- | --- | --- | --- | --- | --- | --- | --- | --- |
|  |  |  |  |  |  | **OR** | **IC** | **P** |
|  |  | **n** | **%** | **n** | **%** |  |  |  |
| Proportion of ambiguous sites (PAS)  (percentile) | <25 | 399 | 23.82 | 87 | 25.59 | 1.10 | 0.84-1.43 | 0.487 |
|  | 25 a <50 | 422 | 25.19 | 76 | 22.35 | 0.85 | 0.64-1.12 | 0.268 |
|  | 50 a <75 | 395 | 23.58 | 81 | 23.82 | 1.01 | 0.77-1.33 | 0.924 |
|  | 75-100 | 459 | 27.40 | 96 | 28.24 | 1.04 | 0.80-1.35 | 0.754 |

Table S7 – Description of the HIV-1 pol sequences included in the phylogenetic analysis.

| Country | Number of sequences | Sampling interval |
| --- | --- | --- |
| Malawi | 5 | 2000-2002 |
| Senegal | 1 | ___ |
| South Africa | 27 | 1999-2014 |
| Zambia | 13 | 1989-2009 |
| Japan | 2 | 2008-2008 |
| Botswana | 7 | 2000-2014 |
| Burundi | 7 | 2002-2007 |
| Tanzania | 2 | 2001-2004 |
| Kenya | 1 | 1998-1998 |
| Uruguay | 1 | 2001-2001 |
| Argentina | 5 | 2001-2004 |
| China | 1 | 2010-2010 |
| Germany | 2 | 2007-2015 |
| Spain | 4 | 2007-2009 |
| United Kingdom | 10 | 2007-2014 |
| India | 2 | 2005-2005 |
| Italy | 7 | 2003-2003 |
| Nepal | 1 | 2016-2016 |
| Peru | 1 | 2009-2009 |
| Poland | 2 | 2001-2002 |
| Portugal | 1 | ___ |
| Sweden | 1 | 2010-2010 |
| Thailand | 1 | 2013-2013 |
| United States of America | 4 | 2001-2012 |
| Brazil | 744 | 1992-2017 |
| Unknown | 2 | ___ |
| Global | 854 | 1992-2017 |
|  |  |  |
|  |  |  |
| Brazilian sequences distributed by state | | |
|  |  |  |
| Region | State | Number of sequences |
| South-East | Espirito Santo (ES) | 1 |
|  | Minas Gerais (MG) | 7 |
|  | Rio de Janeiro (RJ) | 12 |
|  | São Paulo (SP) | 39 |
| South | Paraná (PR) | 109 |
|  | Rio Grande do Sul (RS) | 139 |
|  | Santa Catarina (SC) | 133 |
| North-East | Alagoas (AL) | 1 |
|  | Bahia (BA) | 6 |
|  | Ceará (CE) | 1 |
|  | Maranhão (MA) | 1 |
|  | Paraíba (PB) | 1 |
|  | Piauí (PI) | 1 |
| North | Amazonas (AM) | 6 |
|  | Pará (PA) | 1 |
|  | Rondônia (RO) | 1 |
| Central-West | Goiás (GO) | 3 |
|  | Mato Grosso do Sul (MS) | 6 |
|  | Distrito Federal (DF) | 7 |
| Unknown | ___ | 269 |

Table S8 - Pairwise rates of diffusion between geographic locations using Brazilian state or country outside Brazil where the viral sequence was isolated as discrete trait.

| **Location trait pair** | | **Regions involved** | **BAYES FACTOR** | **POSTERIOR PROBABILITY** |
| --- | --- | --- | --- | --- |
| SC | RS | South | 123895,7829 | 1 |
| SC | SP | South, South-East | 123895,7829 | 1 |
| SC | PR | South | 6513,5858 | 0,9988 |
| SP | MS | South-East, Central-West | 1129,0813 | 0,9933 |
| SP | DF | South-East, Central-West | 251,5648 | 0,9705 |
| SC | MG | South, South-East | 237,2210 | 0,9688 |
| SP | PA | South-East, North | 44,8986 | 0,8545 |
| RJ | BA | South-East, North-East | 42,9874 | 0,8490 |
| SP | RO | South-East, Central-West | 30,6890 | 0,8005 |
| SC | RJ | South, Central-West | 27,0303 | 0,7795 |
| ES | US | International | 16,1299 | 0,6784 |
| RS | GO | South, Central-West | 12,9997 | 0,6296 |
| RS | DE | South, International | 10,8927 | 0,5875 |


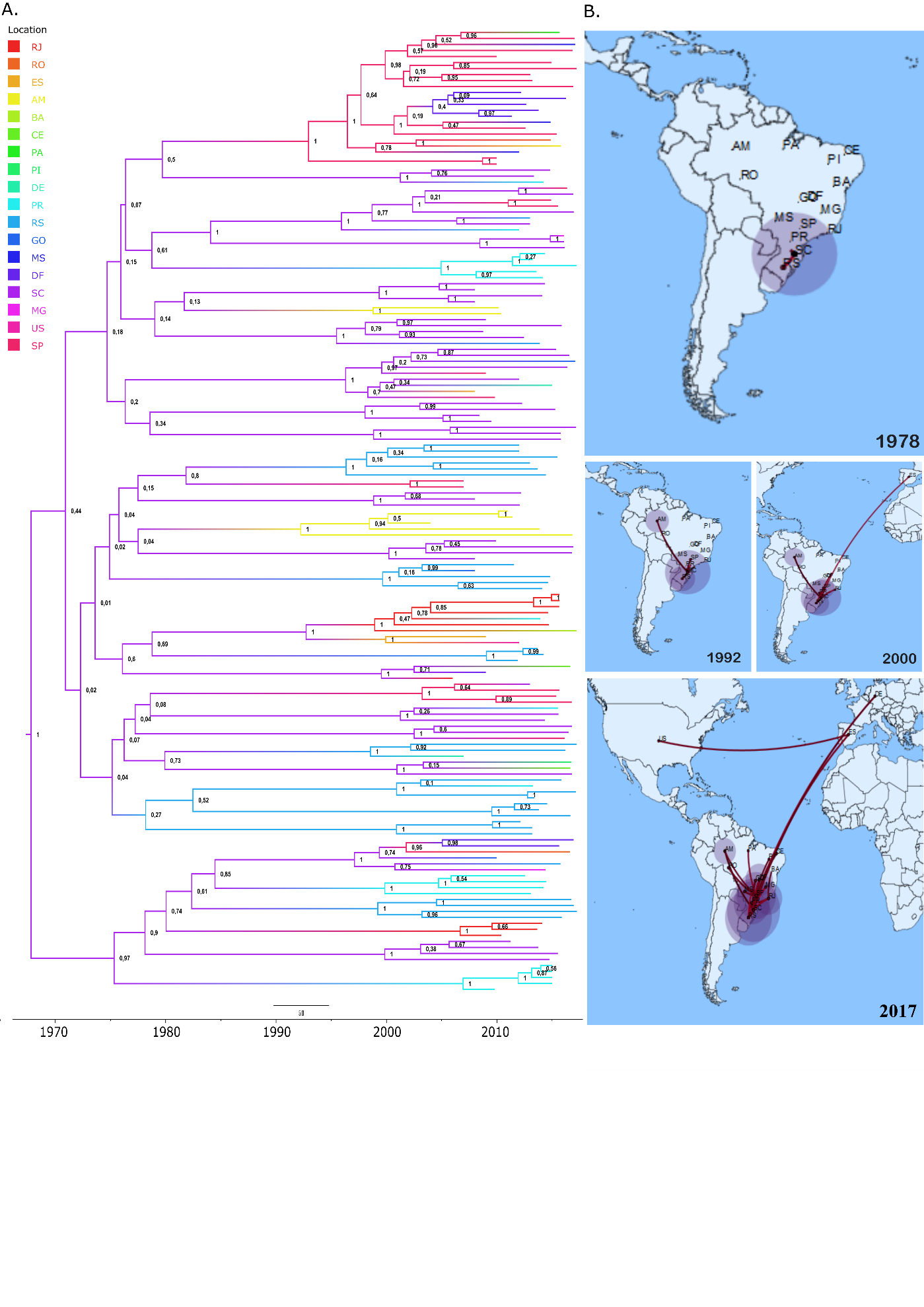


Figure S1. Phylogeographic analysis of the evolution of HIV-1 subtype C transmission clusters. (a) Bayesian MCC time scaled discrete phylogeographic tree built using BEAST v1.10.4 with the Brazilian state or Country outside Brazil where the viral sequence was isolated as the discrete trait. (b) Geographical representation of this transmission history. Acronyms and number of sequencies per region: AM (Amazonas, n=8), BA (Bahia, n=1), CE (Ceará, n=2), DF (Distrito Federal, n=8), GO (Goiás, n=3), MG (Minas Gerais, n=2), MS (Mato Grosso do Sul, n=3), PA (Pará, n=1), PI (Piauí, n=1), PR (Paraná, n=19), RJ (Rio de Janeiro, n=9), RO (Rondônia, n=2), RS (Rio Grande do Sul, n=31), SC (Santa Catarina, n=43), SP (São Paulo, n=23), DE (Germany, n=2), ES (Spain, n=2) and US (USA, n=1). SpreaD3 v0.9.6 (https://rega.kuleuven.be/cev/ecv/software/SpreaD3) was used to visualize the phylodynamic reconstruction resulting from Bayesian inference.
